# Medical-grade buccal swabs versus drugstore cotton swabs: No difference in DNA yield

**DOI:** 10.1016/j.mex.2018.01.006

**Published:** 2018-01-31

**Authors:** Cody A. Ruiz, Morgan E. Chaney, Anthony J. Tosi

**Affiliations:** Department of Anthropology, Kent State University, Kent, OH 44242, United States

**Keywords:** *Buccal cell collection (swab-based)*, Robotic extraction, *Alu* assay, DNA quantitation, Buccal swabs

## Abstract

We tested three types of medical-grade buccal swabs against standard cotton swabs for differences in DNA yield. A panel of swab types – one drugstore (Q-tips**^®^**) and three medical-grade – was used for buccal cell collection from three different individuals. DNA was extracted from all swabs using a QIAcube robot; quantitation values were measured by an *Alu*-based qPCR assay; and differences were compared through a 2-way ANOVA. Our results demonstrate that cotton swabs recover as much DNA as medical-grade swabs, but at a tremendously lower cost. Cotton swabs also display the greatest consistency of DNA yield, as indicated by the lowest coefficient of variation among the four tested swab types. These findings suggest that the use of standard cotton swabs for buccal cell collection offers not only a significant cost savings, but a more consistent method compared to the use of medical-grade swabs.

## Graphical abstract

**Figure:**
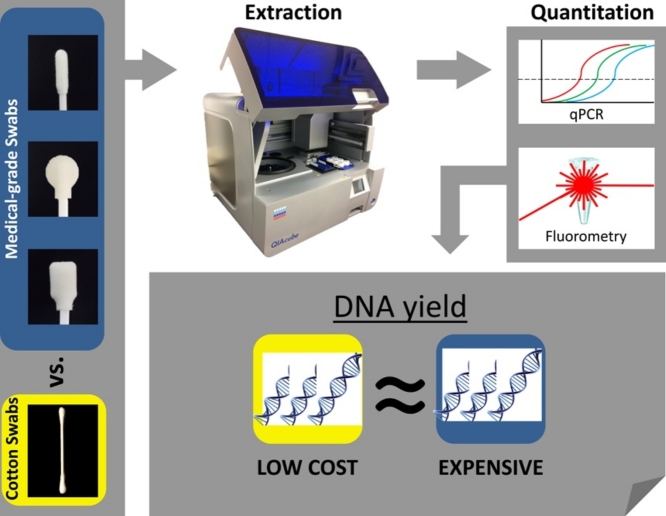

## Method details

The use of buccal swabs is becoming a common method for collecting human and nonhuman DNA samples because it is rapid, non-invasive, and has been shown to produce a sufficient yield for a variety of genetic assays [[1], [2], [3]]. However, there remains a dearth of independent investigation into the DNA yield acquired from various medical and non-medical swab types despite the stark cost difference between the two. This led us to test the hypothesis that medical-grade buccal swabs on average will yield more DNA than standard cotton swabs (Q-tips**^®^**, Unilever, United States).

Following guidelines from established protocols for buccal cell collection [[4], [5]], the three authors provided replicate samples at 24hr intervals by swabbing the inside of the cheek for 15–30 s for each of three medical-grade swab types: Puritan Rectangular Foam Swabs (Cat. #25-1605; n = 15), Puritan Round Foam Swabs (Cat. #25-1805; n = 15), and Puritan HydraFlock**^®^** Flock Swabs (Cat. #25-3306-H; n = 12). These samples were collected from only one cheek since medical-grade swabs have a cotton-tip on only one end. However, when using the standard cotton swabs (Q-tips**^®^**; n = 8) which have two cotton-tipped ends per swab, buccal cells were collected from both cheeks – one end of the swab for each side of the mouth – and the two swab ends were combined as a single sample. Medical-grade swabs were stored in their original packaging and all cotton swabs were stored in paper envelopes at room temperature (25C°) for no more than one week to prevent DNA degradation [1]. DNA extraction was undertaken using the QIAamp DNA Investigator Kit (Cat. #56504) using the Isolation of DNA from Surface and Buccal Swabs Protocol with the QIAcube extraction robot (QIAgen, Germany). Following extraction, total DNA yield was quantitated using the Qubit 3.0 Fluorometer Broad Range DNA assay (Cat. #Q32853) under manufacturer guidelines (Life Technologies, USA). To quantitate only the amount of human DNA, an *Alu* Ya5 subfamily with PerfeCTa^®^ SYBR^®^ Green SuperMix ROX (Cat. #95055-500, Quanta BioSciences, USA) qPCR assay [6] using primers 5′ GTCAGGAGATCGAGACCATCCC 3′ (forward) and 5′ TCCTGCCTCAGCCTCCCAAG 3′ (reverse) was performed on the total DNA samples (StepOnePlus, Life Technologies, USA). We used the statistical program R [7] to analyze Qubit and qPCR values (Table 1).

**Table 1:** Two-way ANOVA Statistics for Swab Type and User-shedding Effects.

Source	df	Sum of Squares	Mean Square	F	Sig.
Swab type	3	203	67.7	0.362	0.781
User-shedding	2	993	496.6	2.654	0.082
Error	44	8234	187.1		
Total	49	9430	751.4		

No significant interaction was detected between the two main effects, allowing us to redirect the df and Sum of Squares from this interaction term back into the linear model. Also, with the non-significant *User-shedding* main effect rolled back into the linear model, the F-value for *Swab type* becomes 0.303 (P > 0.80).

**Fig. 1:**
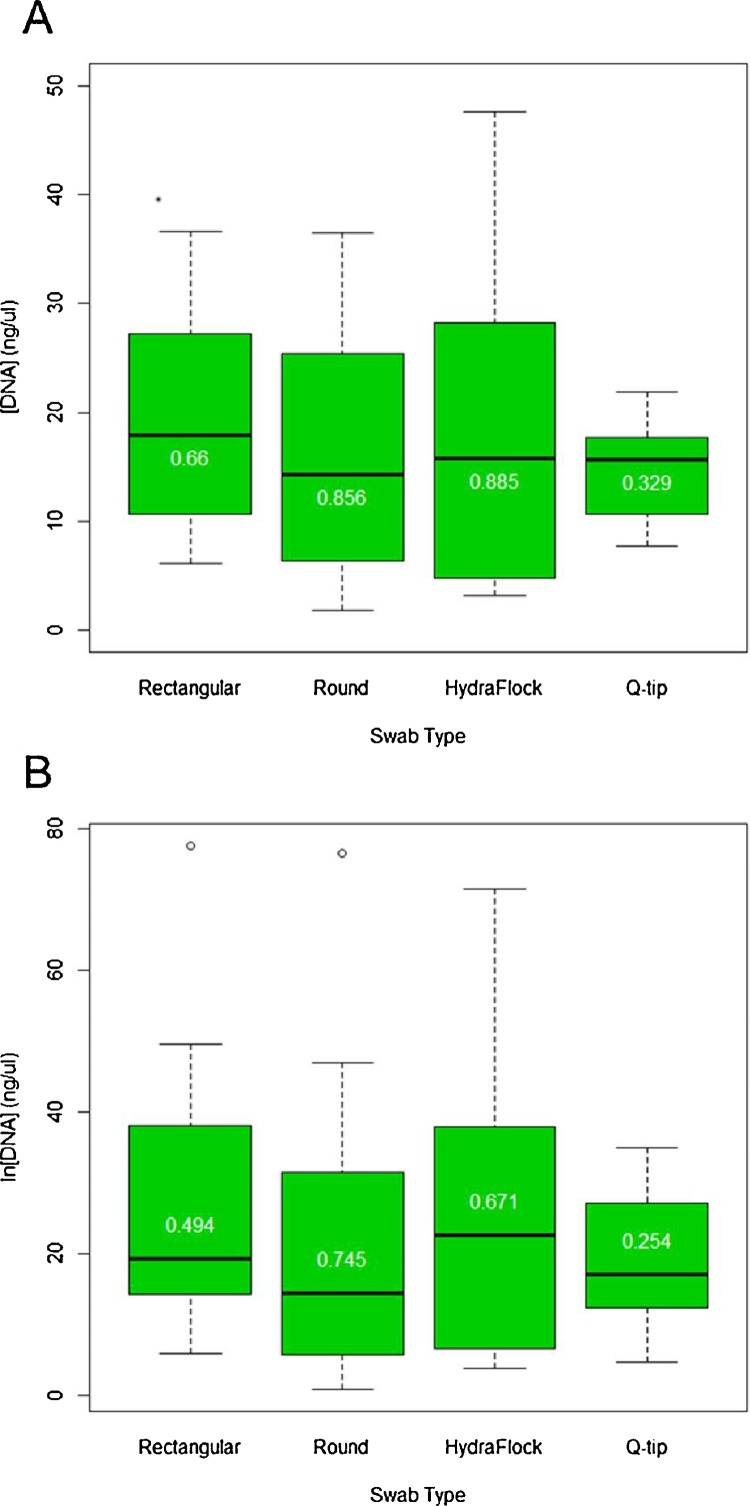
(A) Total DNA quantitation from Qubit 3.0 fluorometer; (B) quantitation of human-specific DNA using Alu Ya5 assay. White numbers within boxplots are coefficients of variation.

We began with a saturated ANOVA that included both swab types and users as factors, in addition to swab type-user interaction term. This was done to control for the tendency of some individuals to shed epithelial cells more readily than others. Comparison of the saturated model to the simple two-way ANOVA (i.e., with only user and swab-type effects) showed no significant interaction between user and swab-type (*F* *=* 1.34, *P >* 0.25). Similarly, comparison of the two-way ANOVA to a one-way ANOVA in which swab-type was the sole independent factor returned no significant difference (*F* *=* 2.71, *P >* 0.05). In this final model, DNA yield still did not vary significantly among the four swab types (one-way ANOVA, *F* *=* 0.303, *P >* 0.80). Furthermore, 95% confidence intervals of the total-DNA extracts showed extensive overlap (Table 2). Moreover, cotton swabs displayed the greatest consistency of DNA yield, as indicated by the lowest coefficient of variation among the four tested swab types (Fig. 1).

**Table 2:** Means and 95% confidence intervals from fluorometer readings (ng/μL).

	Rectangular	Round	HydraFlock	Q-tip
Mean	27.53	21.19	24.58	19.03
Upper 95% CI	37.57	31.96	36.39	24.20
Lower 95% CI	17.48	10.42	12.78	13.85

These data suggest that standard cotton swabs provide a DNA yield equivalent to that of medical-grade swabs. Additionally, cotton swabs are far less than $0.01/swab and present a substantial cost savings when compared to $0.40/HydraFlock^®^-swab, the least expensive medical-grade swab we examined (us.vwr.com/store/catalog/product.jsp?product_id = 7586091). Yet, for work in which *sterile* swabs are critical to maximize defense against contamination (e.g. forensic samples, hospital testing), the substitution of common cotton swabs for medical-grade types may not be feasible [8]. Many research programs, however, have the latitude for this substitution; the chances of a misleading result due to contamination are sufficiently small. In human studies, the number of buccal cells collected onto a cotton swab will likely be much larger than any potentially contaminating cells deposited by mere touch, which tend to yield no more than 1 ng of total DNA [9]. Therefore, a DNA profile of the test subject will override that of the contaminating individual. In nonhuman studies, such contamination is even less of a concern if species-specific primers are employed [10]. Research programs with limited funds, especially in the context of large-scale epidemiological studies or studies where the study population is widely dispersed [11], can therefore stretch their resources by redirecting the associated cost savings to other assays and consumables.

Future comparisons of medical-grade and standard cotton swabs, as used in buccal cell collection, could investigate a number of additional factors; for example, DNA yield may begin to vary with large changes in temperature or storage duration [[12], [13]]. A significant advance in buccal swab use would be the development of a type that specifically harvests human cells as opposed to all cells, including bacterial flora of the mouth or cells of food remains. These sources of exogenous DNA can artificially inflate DNA yield and potentially affect downstream genetic analyses for either cotton swabs or the medical-grade swabs commonly used today.
